# Case Report: Peritoneal and small bowel metastasis from lung adenocarcinoma with EGFR L858R mutation

**DOI:** 10.3389/fonc.2025.1423520

**Published:** 2025-05-13

**Authors:** Tian Tan, Yuerong Huang, Rongxin Yan, Ying Liu

**Affiliations:** Huadu District Peopie's Hospital of Guangzhou, Southern Medical University, Guangzhou, China

**Keywords:** EGFR, lung adenocarcinoma, peritoneal, small bowel metastasis, cancer

## Abstract

Recently, cases of lung cancer metastasizing to atypical sites, such as the small intestine and colon, have been reported; however, peritoneal metastasis remains uncommon. Herein, we present the case of a patient with stage IV lung adenocarcinoma with an EGFR L858R mutation, who developed peritoneal metastasis involving the small intestine, leading to intestinal obstruction and death following osimertinib resistance.

## Introduction

Lung cancer is one of the most common malignant diseases worldwide and a leading cause of cancer-related deaths, accounting for 23.8% of all cancer deaths with 5-year survival rate ranges from 10% to 20% (1). Approximately 40-50% of lung cancer patients present with metastases at the time of diagnosis, commonly affecting lymph nodes, liver, brain, adrenal glands, and bones (2). Recently, there have been reports of lung cancer metastasizing to atypical sites like the small intestine and colon (3); however, peritoneal metastasis remains uncommon (4). Here, we present a case of stage IV lung adenocarcinoma with EGFR L858R mutation that developed peritoneal metastasis involving the small intestine, leading to intestinal obstruction and eventual death following resistance to osimertinib.

### Patient and observation

**Figure 1:**
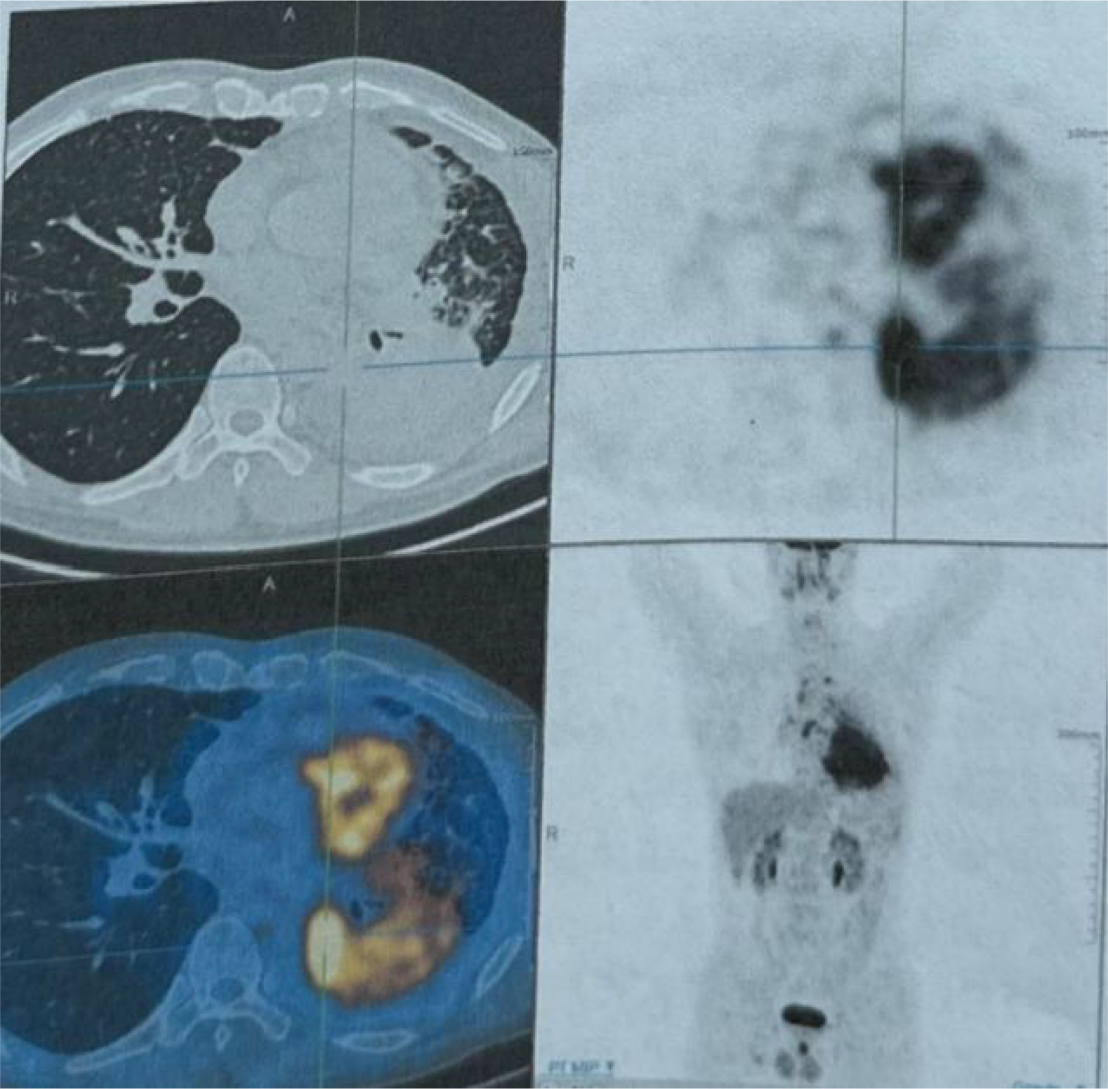
The PET-CT scan conducted on November 24, 2022, revealed a hypermetabolic mass in the left lower lobe of the lung, with a maximum SUV of 12.05 and a maximum transverse diameter measuring approximately 81.0 × 67.6 mm. The mass is indicative of central type lung cancer, accompanied by collapse of the left lung lower lobe and distal obstructive pneumonia. Additionally, there is evidence of lymph node metastasis in the left supraclavicular fossa, mediastinum, left pulmonary hilum, hepatogastric space, and retroperitoneum. Cancerous lymphangitis is observed in the left upper lobe, right middle lobe, and lower lobe, along with left-sided pleural effusion and pericardial effusion.

**Figure 2:**
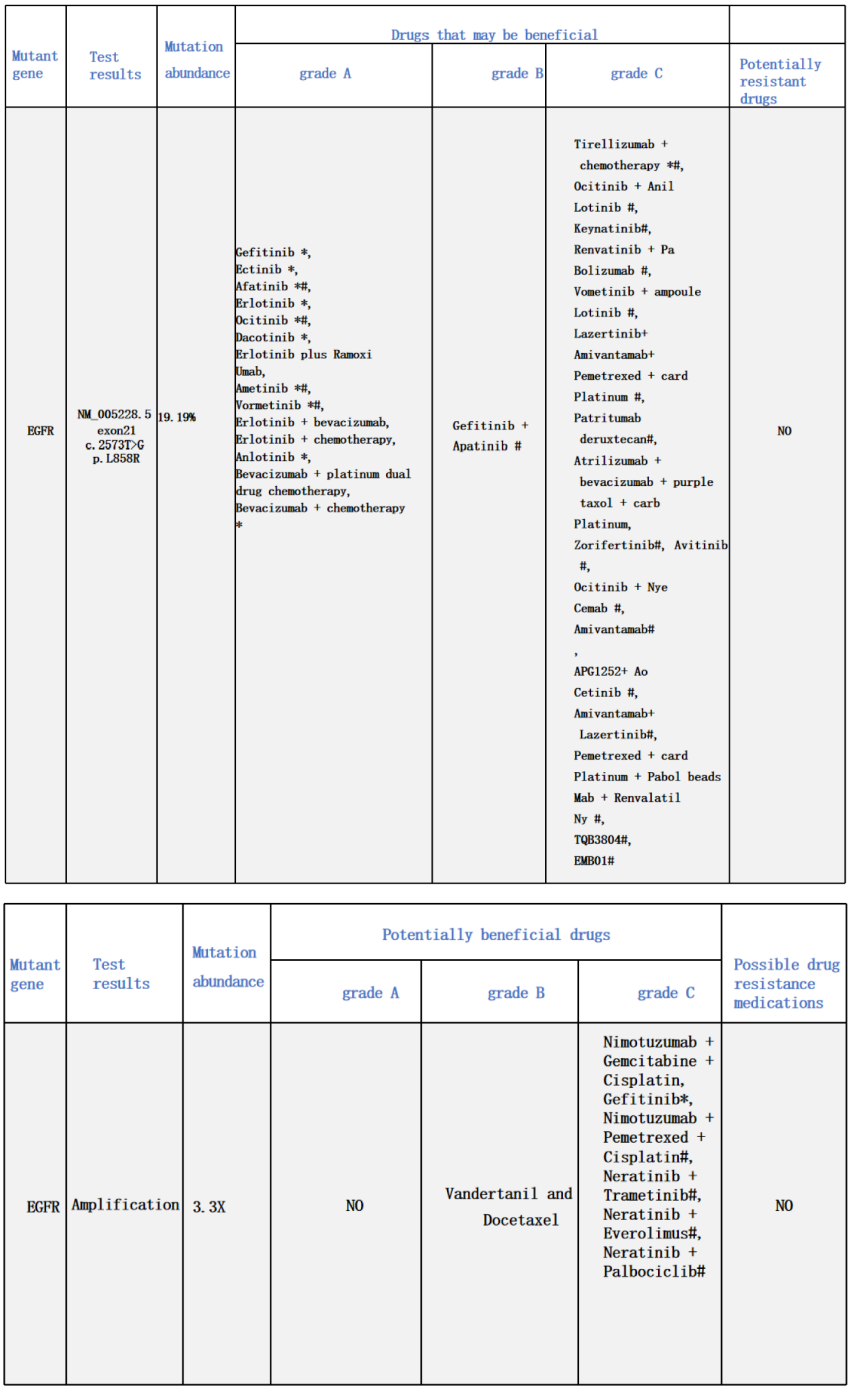
Genetic testing results from December 8, 2022.

**Figure 3:**
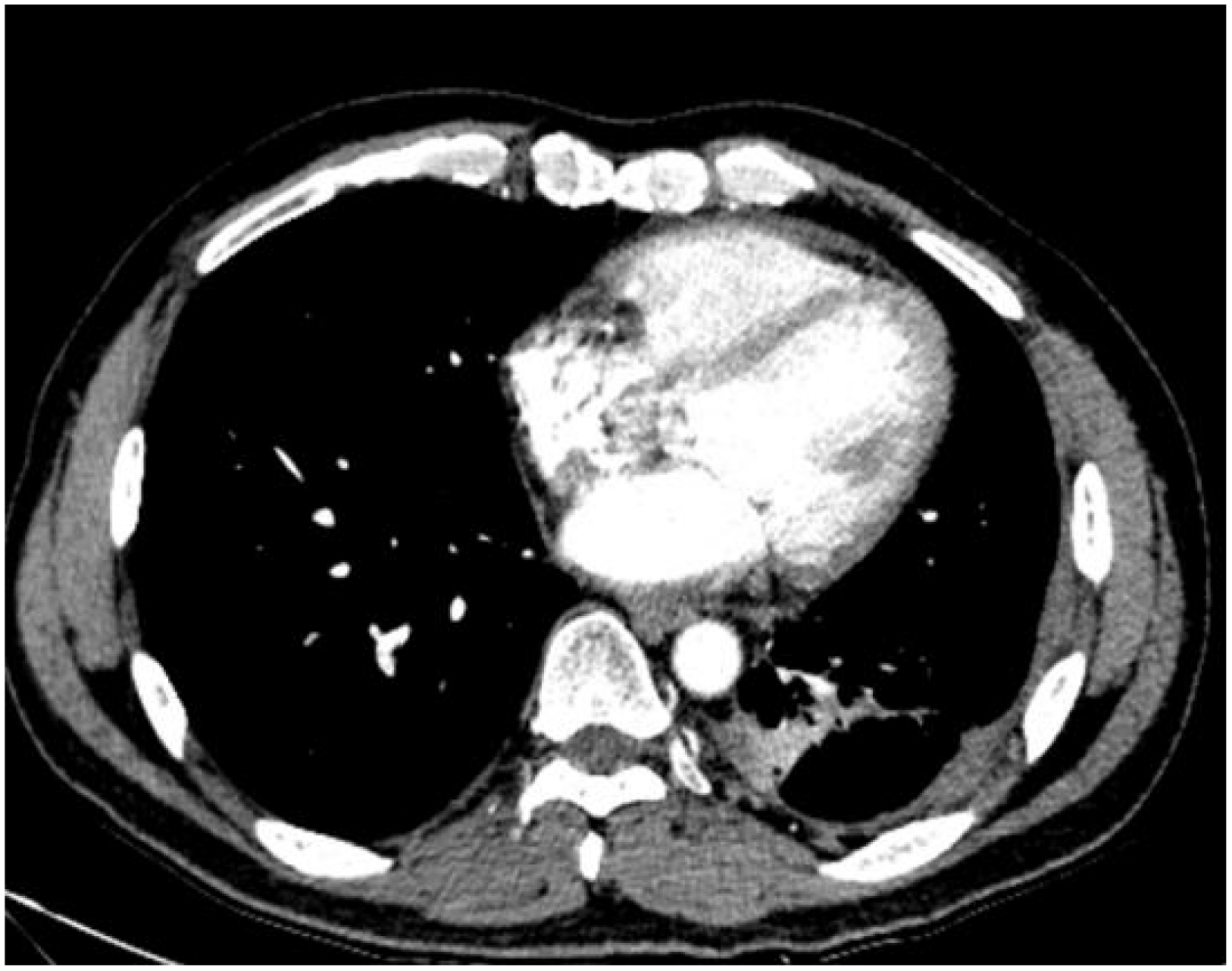
The chest CT scan performed on September 13, 2023, reveals central type lung cancer in the left lower lung with pulmonary metastases, accompanied by cancerous lymphangitis affecting both lungs and improved left lower lung collapse compared to earlier scans. There is also a reduction in metastases to the mediastinal and left pulmonary hilum lymph nodes. Additionally, left pleural metastasis with encapsulating pleural effusion shows improvement compared to previous imaging. A stable solid nodule is noted in the inner basal segment of the right lower lobe, and pericardial effusion has slightly decreased compared to prior examinations.

A 48-year-old male presented with cough and sputum to an external hospital in November 2022. PET-CT imaging revealed a mass (81*68.6mm) with increased glucose metabolism in the left lower lung lobe suspected to be non-central type lung cancer (**Figure 1**). The patient had left lung lower lobe atelectasis, distal obstructive pneumonia, and metastases to multiple sites including left supraclavicular fossa, mediastinum, left pulmonary hilum, hepatogastric space, and retroperitoneal lymph nodes. Cancerous lymphangitis was observed in the left upper, middle, and lower lobes, along with left-sided pleural and pericardial effusions. Bronchoscopy with biopsy showed positive immunohistochemical staining for CK (AE/AE3), TTF-1, CK7, and ki67 (30%), and negative for P40, CK5/6, p63, GATA, and SOX10. Genetic testing revealed an exon 21 L858R mutation (abundance 19.19%), EGFR amplification (abundance 3.3%), TP53 mutation (abundance 14.25%), PMS2 missense mutation, and PD-L1 TPS <1% (**Figure 2**). The diagnosis was left lower lung adenocarcinoma stage T4N3M1a IVA. Osimertinib treatment was initiated on November 16. Regular follow-up examinations showed the best response as partial response (PR) (**Figure 3**).

**Figure 4:**
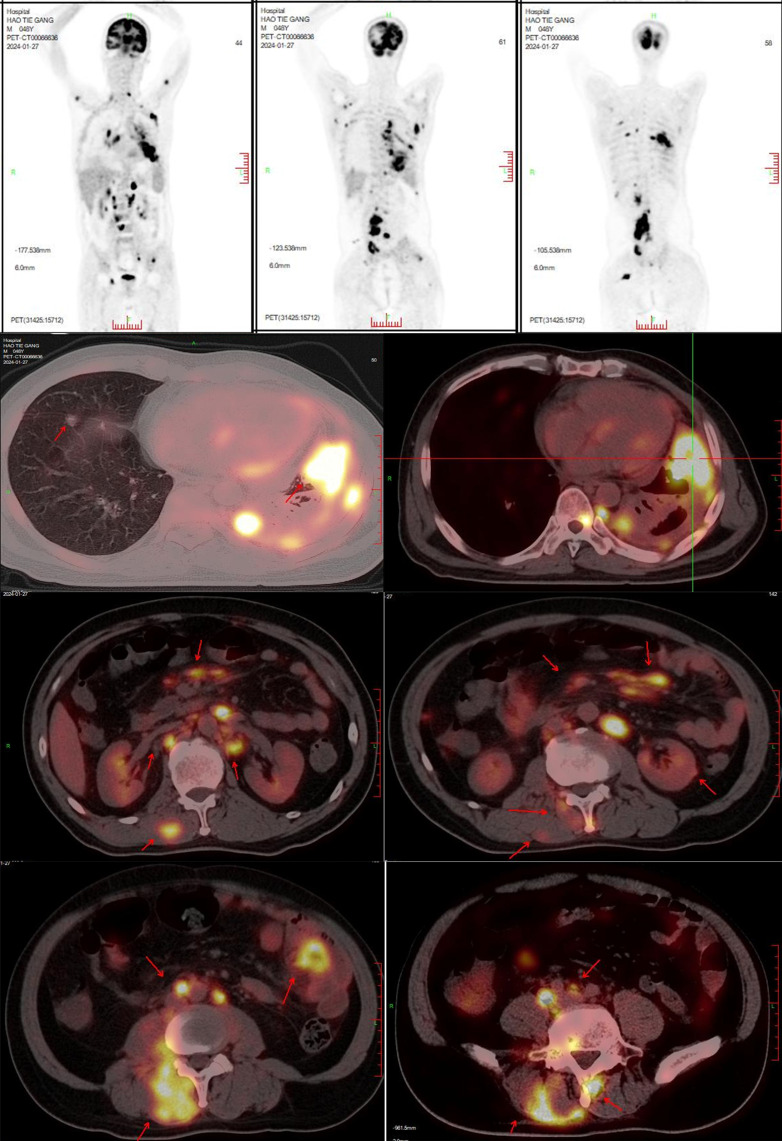
PET-CT on January 27, 2024: 1. Post-treatment findings related to lung cancer:Hypermetabolic lesion resembling residual tumor in the lingular segment of the left upper lobe, involving adjacent pleura. Cancerous lymphangitis in the left upper lobe.Multiple hypermetabolic nodules in both lungs, suggestive of multifocal pulmonary metastases.Uneven thickening of the left pleura with increased glucose metabolism, indicative of pleural metastasis.Left-sided pleural effusion. 2. Multiple hypermetabolic enlarged lymph nodes throughout the body, indicative of multifocal lymph node metastases. 3. Multiple hypermetabolic nodular lesions in the liver, suggestive of multiple hepatic metastases. 4. Bilateral adrenal gland enlargement with increased glucose metabolism, likely representing bilateral adrenal metastases. Multiple hypermetabolic nodular/mass lesions in both kidneys, suggestive of multifocal renal metastases. 5. Multiple nodular and streaky hypermetabolic lesions in the pelvic and abdominal cavities, suggestive of peritoneal metastasis with some lesions involving adjacent intestines. 6. Multiple hypermetabolic nodular/mass lesions between muscles and subcutaneously throughout the body, suggestive of multifocal metastatic lesions. 7. Multiple hypermetabolic lesions indicative of widespread bone destruction, likely representing multiple bone metastases. 8. Hypermetabolic nodular lesion in the right temporal and occipital lobe, suggestive of brain metastasis, accompanied by perilesional edema. 9. Left lower lung lobe collapse with inflammation, and pericardial effusion.

In January 2024, the patient developed lower back and right lower limb pain. A subsequent PET-CT (**Figure 4**) scan on January 27, 2024, showed that 1) Post- treatment residual high metabolic lesions in the lingual segment of the left upper lung lobe, with adjacent pleural involvement; cancerous lymphangitis in the left upper lobe; multiple high metabolic nodules in both lungs suggestive of bilateral lung metastases; uneven thickening of the left pleura with high metabolism indicative of pleural metastasis; and left-sided pleural effusion. 2) Multiple hypermetabolic enlarged lymph nodes throughout the body suggestive of multiple lymph node metastases.

**Figure 5:**
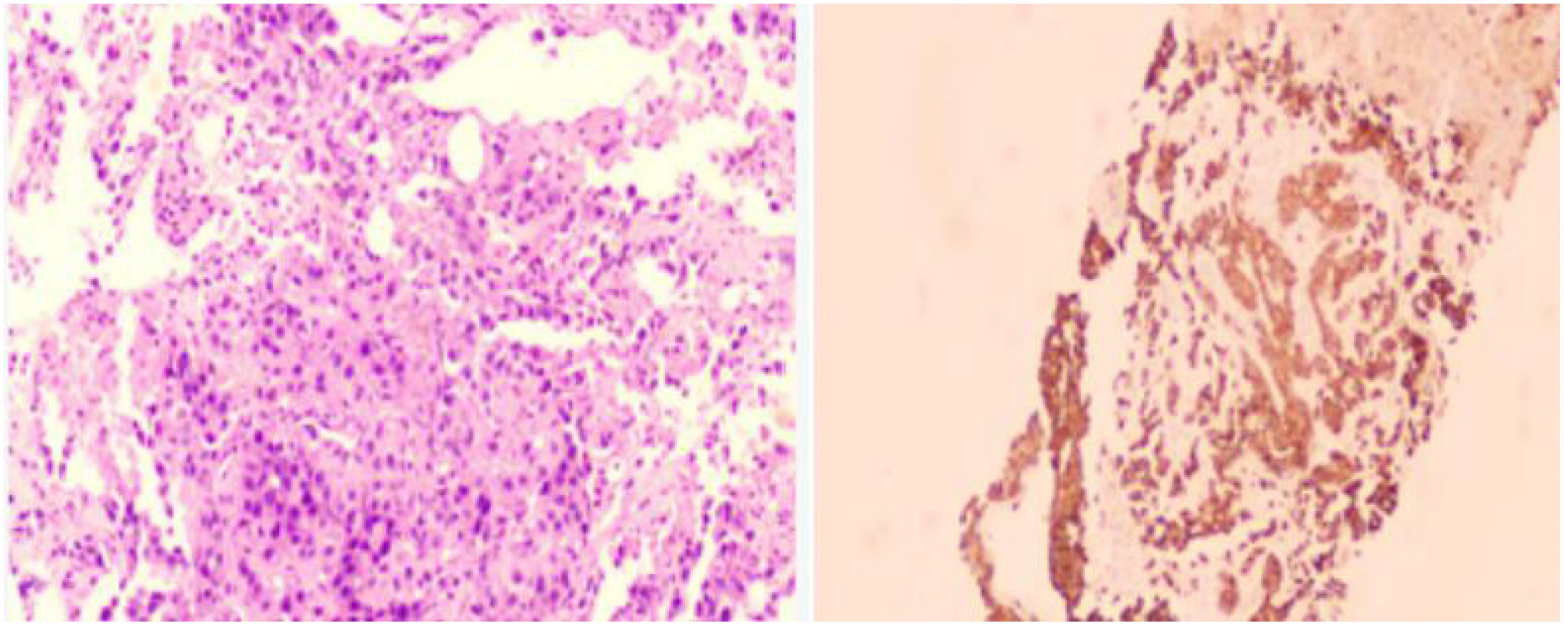
Immunohistochemistry results of the axillary lymph node biopsy conducted on January 29, 2024,:- Positive for CK7, TTF-1, NapsinA- Negative for P40, Syn- Ki67 approximately 35% positive- PD-1 approximately 3% positive- PD-L1 22C3 (tumor proportion score [TPS] = 80%, calculated based on tumor cells only)- Negative for PD-L1 (Neg)Diagnosis of the left axillary lymph node: Metastatic poorly differentiated carcinoma, consistent with an immunohistochemistry profile suggestive of metastatic poorly differentiated adenocarcinoma, possibly of lung origin.

**Figure 6:**
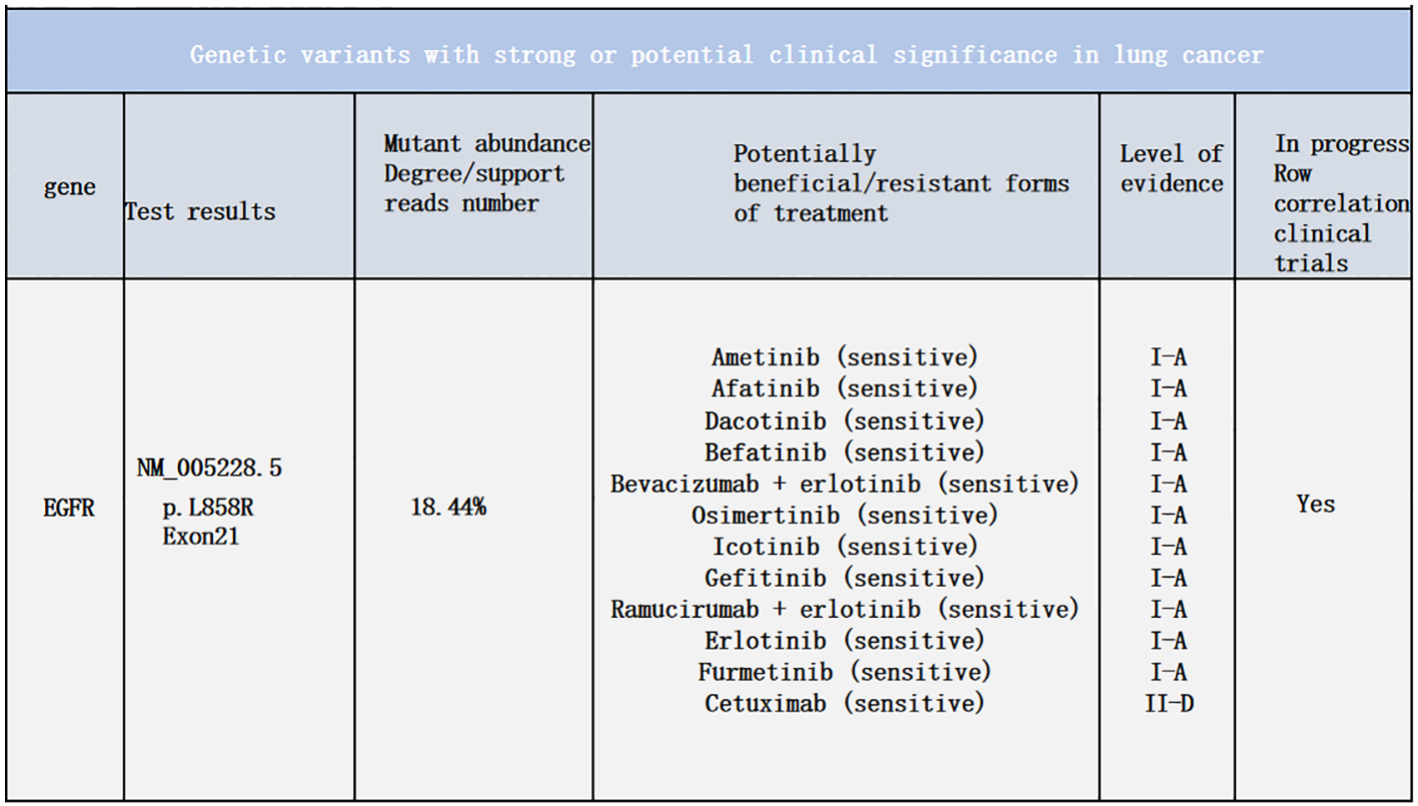
Genetic testing results from February 4, 2024.

**Figure 7:**
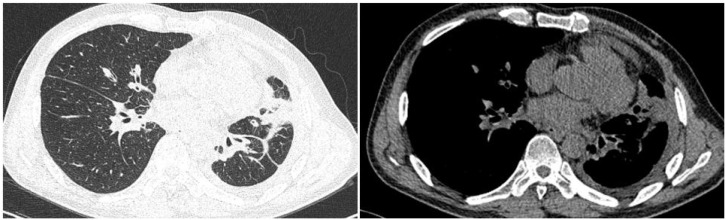
Chest CT scan on March 5, 2024: 1. Considering the medical history, findings consistent with central type lung cancer in the lower lung with pulmonary metastases, along with cancerous lymphangitis in both lungs and left lower lung collapse. There is improvement in mediastinal and left pulmonary hilum lymph node metastases, as well as left pleural metastasis with encapsulating pleural effusion. 2. Multiple solid nodules in both lungs, suggestive of metastatic tumors, showing reduction compared to previous scans.

3) Multiple nodular high metabolic lesions in the liver suggestive of multiple liver metastases. 4) Bilateral adrenal gland thickening with increased metabolism suggestive of bilateral adrenal metastases; multiple nodular/high metabolic lesions in both kidneys indicative of bilateral kidney metastases. 5) Multiple nodular/striated high metabolic lesions in the pelvic and abdominal cavities indicative of peritoneal metastases, some lesions affecting adjacent intestines. 6) Multiple nodular/high metabolic lesions in muscles and subcutaneous tissues throughout the body indicative of multiple metastases.7) Multiple sites of bone destruction with increased metabolism suggestive of multiple bone metastases. 8) Nodular high metabolic lesions in the right temporoparietal lobe suggestive of brain metastasis with surrounding edema. 9) Left lung lower lobe atelectasis with inflammation and pericardial effusion were noted. Disease progression was indicated with a progression-free survival (PFS1) of 14 months. Following lymph node aspiration from the left axilla, metastatic poorly differentiated adenocarcinoma positive for TTF-1, ki67 (35%), CK7, and negative for p40, Syn, SOX10 was identified, indicating lung origin (**Figure 5**). Despite disease progression and a performance status score of 2, repeat genetic testing confirmed the persistence of the exon 21 L858R mutation (abundance 18.44%) (**Figure 6**). From January 31 to February 21, 2024, the patient received chemotherapy with bevacizumab 400mg, albumin paclitaxel 300mg, and cisplatin 30mg on days 1-2, while osimertinib was increased to 160mg. Subsequent chest CT on March 5, 2024, showed improvement in left pleural metastasis and encapsulated pleural effusion, with fewer solid nodules in both lungs indicating reduced metastases. An abdominal CT scan on March 5, 2024 (**Figure 7**), revealed peritonitis and suspected peritoneal metastasis, with partial small bowel obstruction and ilea segment thickening suggestive of inflammatory changes. Conservative treatment with antibiotics, gastrointestinal decompression, and enemas alleviated abdominal symptoms; however, the patient’s performance status declined, rendering chemotherapy intolerable. On March 5, 2024, anlotinib 10mg was initiated, and continued for 12 days. Due to persistent lower back pain. Local radiotherapy was performed at an external hospital to manage pain, although intestinal obstruction symptoms progressively worsened. Consultations with interventional radiology and gastrointestinal surgery advised conservative management. Unfortunately, the patient passed away on March 30, 2024, 63 days following the diagnosis of peritoneal metastasis, with an overall survival (OS) of 16 months from the initial diagnosis.

## Discussion

The occurrence of symptomatic intestinal metastasis from lung cancer is extremely rare in clinical practice. The first documented case of lung squamous cell carcinoma metastasizing to the small intestine and causing intestinal perforation was reported in 1980 (5). A retrospective analysis by French scholars in 1999 examined 1,399 lung cancer patients who underwent surgical intervention for intestinal metastasis between 1984 and 1996, revealing that only 0.5% (7/1399) of these patients exhibited symptomatic small bowel metastasis (6).

However, evidence suggests that the incidence of this tumor type may be higher than clinically appreciated. A review of autopsy data from 1982 encompassing 423 cases of primary lung tumors over 36 years identified gastrointestinal metastases in 58 cases (14%). The most commonly affected site was the esophagus (33/58, 56%), followed by small bowel infiltration (20/58, 34%), with 6 cases experiencing complications such as perforation and peritonitis due to metastatic lesions. The predominant histological types observed were squamous cell carcinoma (19 cases, 33%), large cell carcinoma (17 cases, 29%), and oat cell carcinoma (11 cases, 19%). Another type of bowel obstruction is chronic intestinal pseudo-obstruction (CIPO), which was reported as the rare para-neoplastic syndromes of small cell lung cancer. CIPO can be due to a dysfunction in coordinated propulsive activity in the gastrointestinal tract, with clinical features of mechanical small bowel obstruction (7). In this particular case, the histological type was adenocarcinoma with an EGFR mutation, and current literature does not report any association between the genetic subtype of lung cancer and the occurrence of intestinal metastasis (8).

Peritoneal surface malignancies (PSM) comprise a heterogeneous group of quite different cancers, and all of cancers are unique in their proclivity for peritoneal dissemination. Ovarian cancer and gastric cancer are the most common primary tumors for peritoneal metastasis (9).Peritoneal metastasis originating from lung cancer is considered rare and is typically identified primarily through autopsies (10). Recent advancements in imaging techniques such as CT scans and PET-CT have enhanced the accuracy of diagnosing peritoneal carcinomatosis. In this case, both CT and PET-CT scans detected peritoneal metastasis, with PET-CT exhibiting greater sensitivity in detecting small bowel lesions compared to conventional CT scans (11). In a study by Satoh in 2001 (12), analyzing 1,041 lung cancer patients over 26 years, 12 cases (1.2%) were identified with peritoneal metastasis during their clinical course. Patients with histological types of large cell carcinoma and adenocarcinoma exhibited a higher incidence of peritoneal metastasis. Among these cases, 6 patients had intraperitoneal metastasis, and 9 had pleural metastasis. Tejas Patil (13) conducted an analysis at the University of Colorado Cancer Center involving 410 diagnosed cases of metastatic non-squamous non-small cell lung cancer, with 8% (33/410) of patients experiencing peritoneal metastasis. Malignant pleural disease showed a strong association with subsequent peritoneal spread in patients with peritoneal metastasis. However, no correlation was observed between oncogene status and peritoneal carcinomatosis. In this case, the patient initially received a diagnosis of pleural metastasis, which may have contributed to the development of peritoneal metastasis. Nevertheless, not all patients with pleural metastasis progress to peritoneal metastasis, suggesting that additional factors play a role in the spread to the peritoneum.

Symptomatic metastasis to the small intestine may necessitate surgical management, involving resection of the affected segment and potentially creating a primary enterostomy. However, the prognosis associated with this condition is generally very poor. Despite advancements in medical and surgical approaches, the management of peritoneal carcinomatosis remains predominantly palliative, with a median survival typically not exceeding 2 months (14). Literature reports show perioperative mortality rates ranging from 60% to 100%. In a retrospective study by French scholars, six patients died within 8 months after metastasectomy, with one surviving for 22 months post-small bowel resection but subsequently developing brain metastasis requiring two surgeries (15). Some researchers have documented cases of early-stage lung cancer patients undergoing emergency debulking surgery for diffuse peritoneal metastasis to alleviate intestinal obstruction, achieving favorable outcomes and long-term survival (16, 17). However, this approach necessitates stringent patient selection based on tumor control status, performance status (PS) score, and other factors. For SCLC-associated CIPO, Mohammad reported a case of a middle-aged patient involved good clinical and radiological response to systemic anticancer therapy in form of combination of chemo-immunotherapy (18). In this particular case, despite employing second-line chemotherapy and anti-angiogenic drugs resulting in lung lesion shrinkage, peritoneal metastasis continued to progress, ultimately leading to the patient’s death.

## Conclusion

In summary, peritoneal metastasis from lung cancer leading to small intestine obstruction is a rare and challenging occurrence. Lung cancer patients with unexplained abdominal symptoms during treatment should undergo appropriate diagnostic evaluations. Early and rigorous selection for surgical intervention could potentially extend patient survival.

## Data Availability

The original contributions presented in the study are included in the article/supplementary material. Further inquiries can be directed to the corresponding author.
